# MnO_2_ Nanoflowers Induce Immunogenic Cell Death under Nutrient Deprivation: Enabling an Orchestrated Cancer Starvation‐Immunotherapy

**DOI:** 10.1002/advs.202002667

**Published:** 2020-12-31

**Authors:** Yannan Yang, Zhengying Gu, Jie Tang, Min Zhang, Yang Yang, Hao Song, Chengzhong Yu

**Affiliations:** ^1^ Australian Institute for Bioengineering and Nanotechnology The University of Queensland St. Lucia Brisbane QLD 4072 Australia; ^2^ School of Chemistry and Molecular Engineering East China Normal University Shanghai 200241 P. R. China

**Keywords:** autophagy, immunogenic cell death, immunotherapy, MnO_2_ nanoparticles

## Abstract

MnO_2_ nanoparticles have been widely employed in cancer immunotherapy, playing a subsidiary role in assisting immunostimulatory drugs by improving their pharmacokinetics and/or creating a favorable microenvironment. Here, the stereotype of the subsidiary role of MnO_2_ nanoparticles in cancer immunotherapy is challenged. This study unravels an intrinsic immunomodulatory property of MnO_2_ nanoparticles as a unique nutrient‐responsive immunogenic cell death (ICD) inducer, capable of directly modulating immunosurveillance toward tumor cells. MnO_2_ nanoflowers (MNFs) constructed via a one pot self‐assembly approach selectively induce ICD to nutrient‐deprived but not nutrient‐replete cancer cells, which is confirmed by the upregulated damage associated molecular patterns in vitro and a prophylactic vaccination in vivo. The underlying mechanism of the MNFs‐mediated selective ICD induction is likely associated with the concurrently upregulated oxidative stress and autophagy. Built on their unique immunomodulatory properties, an innovative nanomaterials orchestrated cancer starvation‐immunotherapy is successfully developed, which is realized by the in situ vaccination with MNFs and vascular disrupting agents that cut off intratumoral nutrient supply, eliciting potent efficacy for suppressing local and distant tumors. These findings open up a new avenue toward biomedical applications of MnO_2_ materials, enabling an innovative therapeutics paradigm with great clinical significance.

## Graphical abstract

An intrinsic immunomodulatory property of MnO_2_ flowers in regulating the immunogenicity of cancer cells in a nutrient‐responsive manner is unraveled. This finding leads to an innovative nanomaterials orchestrated cancer starvation‐immunotherapy by co‐administering MnO_2_ nanoflowers and vascular disrupting agents that suspend intratumoral nutrient supply, realizing an “in situ vaccination” approach with remarkable antitumor performance in suppressing local and distant tumors.

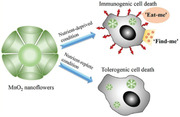

## 1 Introduction

Immunotherapy that harnesses the immune system to eradicate tumor cells is a remarkable breakthrough in the combat against cancer.^[^ ^1^ ^]^ One typical approach to activate the immune system is through the induction of immunogenic cell death (ICD),^[^ ^2^ ^]^ which not only directly kills cancer cells, but also results in highly exposed damage associated molecular patterns (DAMPs) on tumor cells to recruit immune cells and stimulate antitumor immunity.^[^ ^3^ ^]^ To date, a library of ICD inducers have been identified, including certain chemodrugs and photosensitizers,^[^ ^4^ ^]^ whose immunotherapeutic efficacy can be significantly improved with the assistance of nanoparticles.^[^ ^5^ ^]^ One typical example is MnO_2_ nanoparticles, whose diverse functionalities, such as longitudinal (T1) relaxation, glutathione (GSH) oxidation, enzyme mimicking, and Fenton‐like activity, have been well‐studied.^[^ ^6^ ^]^ Benefiting from those interesting properties, MnO_2_ nanoparticles could improve the efficacy of ICD inducers mainly through the following three manners, including i) serving as traceable drug carriers to enable enhanced cellular uptake and controlled drug release, ii) serving as oxidant to deplete GSH to promote the oxidative damage, and iii) serving as catalase to decompose H_2_O_2_ into molecular oxygen to improve photodynamic efficiency.^[^ ^7^ ^]^ As such, MnO_2_ nanoparticles primarily played a subsidiary role to assist immunostimulatory drugs by improving their pharmacokinetics and/or creating a favorable microenvironment. However, their intrinsic immunomodulatory activity in directly eliciting antitumor immunity remains largely unexplored.

Cancer starvation therapy is another attractive strategy in treating malignant tumors,^[^ ^8^ ^]^ which can be realized by applying vascular disrupting agents (VDAs) to selectively damage the tumor vasculature system to block nutrient supply, and thus cause tumor cell necrosis.^[^ ^9^ ^]^ However, its efficacy is dramatically hindered by the surviving peripheral tumor rim remained post‐treatment, which is a major cause of tumor regrowth and metastasis.^[^ ^10^ ^]^ Given the robust efficacy of antitumor immunity particularly in controlling the residue and spreading tumor cells, it is expected that integrating immunotherapy with cancer starvation would be highly promising to render potent anti‐cancer performance.^[^ ^11^ ^]^ To this goal, developing therapeutic agents to achieve their orchestration would be of critical importance, yet rarely reported to our knowledge.

In this work, a “hidden” intrinsic activity of MnO_2_ nanomaterials in modulating the immunogenicity of cancer cells in a nutrient‐responsive manner is unraveled. It was shown that MnO_2_ nanoflowers (MNFs) synthesized via a one‐pot self‐assembly approach can efficiently induce ICD to cancer cells under nutrient‐deprived but not nutrient‐replete conditions. This finding was supported by the significantly increased exposure of DAMPs of MNFs treated starved cells in vitro, and by a prophylactic vaccination model in vivo. The underlying mechanism was attributed to the concurrent induction of oxidative stress and autophagy by MNFs in nutrient‐deprived cancer cells. Built on the discovery of the unique nutrient‐dependent ICD‐inducing activity of MNFs, an innovative nanoparticles‐mediated synergistic cancer starvation‐immunotherapy based on in situ vaccination with combined MNFs and a VDA, 5,6‐dimethylxanthenone‐4‐acetic acid (DMXAA) was demonstrated, showing remarkable antitumor efficacy in a bilateral breast cancer tumor model.

## 2 Results and Discussion

### 2.1 Synthesis and Characterization of MNFs

**Figure 1:**
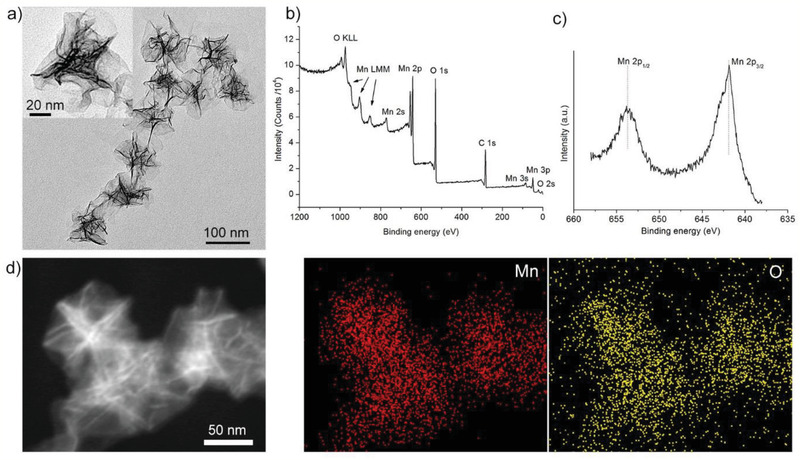
Characterization of MNFs. a) TEM images with different magnifications. b) XPS survey. c) High resolution XPS spectra of Mn. d) Dark‐field STEM and corresponding elemental mapping.

The MNFs were self‐assembled through a redox reaction between potassium permanganate (KMnO_4_) and 2‐(*N*‐morpholino)ethanesulfonic acid (MES). This reaction system enabled rapid synthesis (within 30 min) of uniform MNFs, which have previously been reported with high reactivity in bio‐systems.^[^ ^6g^ ^]^ As shown in transmission electron microscopy (TEM) image (**Figure** **1**a), MNFs exhibit a flower‐like morphology with average particle size of ≈94 nm and wall thickness of ≈3 nm. The extremely thin wall of MNFs implies that they were assembled by nanosheets. Previous reports indicated that, depending on the synthetic system and parameters, the MES/KMnO_4_ system could produce either 2D^[^ ^6^, ^12^ ^]^ or 3D^[^ ^6^, ^13^ ^]^ nanosheets. In the present work, the concentrations of KMnO_4_ and MES were optimized to reduce the particle size below 100 nm to make them suitable for bio‐applications. The chemical composition of MNFs was characterized by X‐ray photoelectron spectroscopy (XPS), showing the presence of Mn and O (Figure 1b). High resolution XPS spectra (Figure 1c) show the binding energy difference of Mn 2p_1/2_ and Mn 2p_3/2_ of 11.6 eV, indicating a Mn^4+^ valent state.^[^ ^14^ ^]^ The homogeneous distribution of Mn and O was revealed by scanning TEM (STEM) and the corresponding energy‐dispersive X‐ray spectroscopy (EDS) mapping results (Figure 1d). According to the X‐ray diffraction (XRD) pattern of the as‐prepared MNFs, there was no characteristic peaks assigned to any crystalline form of MnO_2_, suggesting that the product is nearly amorphous (Figure S1a, Supporting Information). These results collectively confirm the successful formation of MNFs with an amorphous nature.

To improve the colloidal stability of as‐prepared MNFs in aqueous phase, polyethylene glycol (PEG) modification was applied (refer to method section for details). In contrast to aggregated bare MNFs, the PEGylated MNFs showed greatly improved dispersity in phosphate buffer saline (PBS) (Figure S1b, Supporting Information). This was further confirmed by the dynamic light scattering (DLS), showing a hydrodynamic size of ≈126 nm with a narrow size distribution for PEGylated MNFs (Figure S1c, Supporting Information), while the hydrodynamic size of bare MNFs was too large to be detected by DLS (beyond 10 µm due to severe aggregation). The surface charge of bare MNFs was altered from −27.3 (due to the presence of surface Mn‐O^−^) to a near neutral charge of −8.2 mV in PEGylated MNFs (Figure S1d, Supporting Information), suggesting successful PEGylation. The PEG content modified on MNFs was 7.6 ± 1.2% according to elemental analysis. The abbreviation of “MNFs” used hereafter denotes PEGylated MNFs.

### 2.2 Cancer Cell and Tumor Inhibition Activity

**Figure 2:**
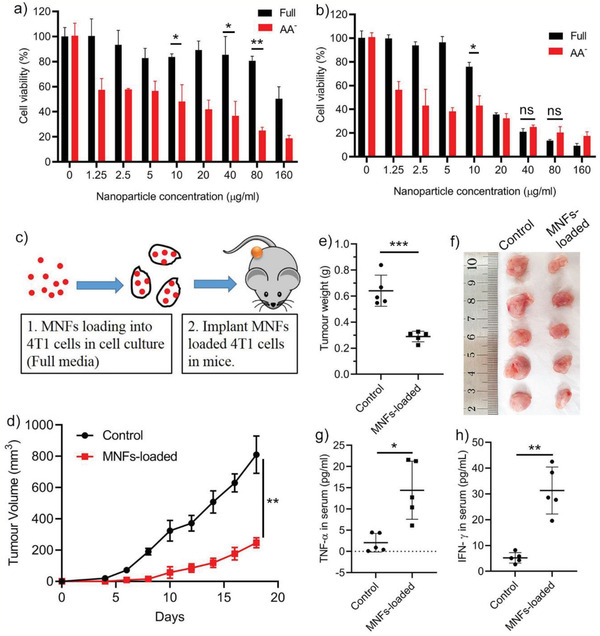
Low dose MNFs remarkably suppress tumorigenic activity of cancer cells. a,b) Viability of 4T1 cancer cells treated by various concentration of MNFs at 6 h (a) and 24 h (b). c) Schematic illustration on the workflow. 4T1 cells treated with MNFs nanoparticles (10 µg mL^−1^) in full media for 6 h before creating xenografts in Balb/c mice. d) Tumor growth profile. e) Tumor weight measured on day 18. f) The optical images of tumors collected on day 18. g,h) Cytokine levels in the serum measured on day 10. Data represent mean ± S.D. (*n* = 4 for (a), (b); *n* = 5 for (d)–(h). Unpaired *t*‐test (for a, b, e, g, h) and one‐way ANOVA (for d) were used to determine statistical differences. (*p < 0.05, **p < 0.01, ***p < 0.001, ns: not significant).

The cytotoxicity of MNFs in 4T1 breast cancer cells was firs investigated under nutrient‐replete (with amino acids) and nutrient‐deprived (without amino acids) culture conditions. At incubation time of 6 h, MNFs were well‐tolerated by cells in nutrient‐replete (full) culture media up to a dosage of 80 µg mL^−1^ (80.6% cell viability). In contrast, MNFs showed significantly enhanced toxicity to cells cultured in amino‐acid‐deprived (AA^−^) media as demonstrated by the dramatically reduced cell viability even at a concentration as low as 1.25 µg mL^−1^ (**Figure** **2**a). At prolonged incubation time of 24 h, MNFs with concentration below 10 µg mL^−1^ still showed higher cytotoxicity to cells cultured in AA^−^ media than that cultured in full media (Figure 2b). Interestingly, this trend was reversed when the MNFs dosages were above 40 µg mL^−1^. We initially speculated that the reduced sensitivity of starved cancer cells to high concentration of MNFs at 24 h could be due to the compromised endocytosis as a result of insufficient nutrient supply. However, time‐resolved quantification of cellular uptake through inductively coupled plasma‐optical emission spectrometry indicated that the difference in cellular uptake of MNFs in two conditions (full and AA^−^ media) was minor (Figure S2a, Supporting Information). Alternatively, the activation of autophagy could be another possible reason, which will be discussed later. Collectively, these results clearly indicate that nutrient‐deprived cancer cells are highly sensitive to low dosage MNFs treatment.

To validate this finding at in vivo condition, a modified tumor xenografts model was utilized. We incubated 4T1 cells with 10 µg mL^−1^ MNFs in full media, which had little effect on cell viability (Figure 2a), and then inoculated MNFs‐treated cells, as well as untreated cells (Figure S2b, Supporting Information), into mice as tumor xenografts. It is known that the inoculated cancer cells would initially experience a nutrient‐deprived state due to the lack of vasculature.^[^ ^15^ ^]^ Therefore, this type of tumor xenograft created by pre‐incubating cancer cells with nanoparticles has been used as a model to provide nutrient deprivation condition in vivo at very early stage, that is, before the formation of detectable solid tumors.^[^ ^16^ ^]^ MNFs loaded 4T1 cells exhibited significantly delayed growth profile compared to control cells (Figure 2d–f; Figure S3, Supporting Information), and the tumorigenesis was not detected up to 8 days post injection. These results confirmed that MNFs at low concentrations can be toxic to starved cancer cells not only in vitro, but also in vivo. However, considering the moderate cell inhibition activity of MNFs at 10 µg mL^−1^ (≈43% cell viability) under a completely amino acid free condition in vitro (Figure 2b), the highly potent tumor inhibition activity of MNFs, particularly within the first 8 days, was somewhat surprising. We therefore speculated that, apart from the cytotoxicity of MNFs, the in situ generated immune response could be another mechanism that contributed to the tumor inhibition performance. Indeed, TNF‐*α* and IFN‐*γ* levels, two typical Th1 cytokines, in the serum of mice implanted with MNFs loaded 4T1 cells were significantly elevated (Figure 2g,h), implying the occurrence of immune response.

### 2.3 MNFs Selectively Induce ICD in Nutrient‐Deprived Cancer Cells

**Figure 3:**
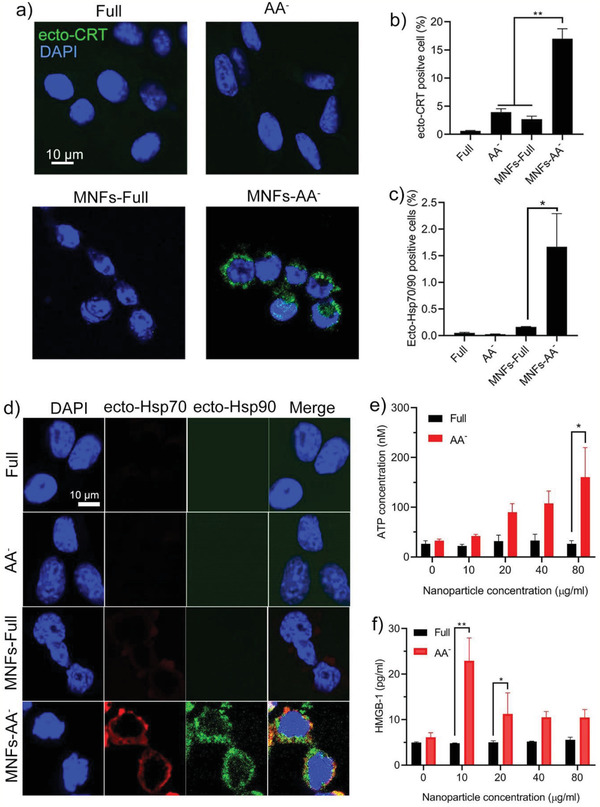
MNFs induce immunogenic cell death in AA^−^ conditions. a) CLSM image of the surface exposure of ecto‐CRT on 4T1 cells. b) Surface exposure of ecto‐CRT on 4T1 cells measured by flow cytometry. c) Surface exposure of ecto‐Hsp70 and ecto‐Hsp90 on 4T1 cells measured by flow cytometry. d) CLSM image of the surface exposure of ecto‐Hsp70 and ecto‐Hsp90 on 4T1 cells. e,f) Secretion of ATP and HMGB‐1 in the cell culture media. Data represent mean ± S.D. (*n* = 3). Unpaired *t*‐test was used to determine statistical differences. (*p < 0.05, **p < 0.01, ***p < 0.001).

We next sought to understand when (before or after implantation) and how the in situ immune response observed above was generated. It has been well‐documented that the exposure of DAMPs largely dictate the immunogenicity of cancer cells, thus their exposure level was measured on MNFs treated cells in full or AA^−^ culture media. Calreticulin (CRT) is one typical DAMP to characterize ICD, since it can translocate from endoplasmic reticulum to cell external surface under stressed condition, acting as a “eat me” signal to facilitate the engulfment of dying cells by antigen presenting cells.^[^ ^17^ ^]^ Cells treated by MNFs in AA^−^ culture media (MNFs‐AA^−^) exhibited significantly increased exposure level of ecto‐CRT as visualized by confocal laser scanning microscopy (CLSM) image (**Figure** **3**a). The ecto‐CRT level of MNFs‐AA^−^ cells was quantified by flow cytometry (Figure 3b and Figure S4a, Supporting Information), showing a ≈17% ecto‐CRT positive population, while AA^−^ media alone or MNFs in full media (MNFs‐Full) failed to induce significant ecto‐CRT exposure. Moreover, heat shock protein 70 (Hsp70) and heat shock protein 90 (Hsp90) are two recently discovered “eat me” signals.^[^ ^18^ ^]^ Cells under MNFs‐AA^−^ condition showed a more than tenfold increase of co‐exposure levels of ecto‐Hsp70/ecto‐Hsp90 compared to other groups (Figure 3c and Figure S4b, Supporting Information). CLSM image clearly confirmed the location of Hsp70 and Hsp90 proteins on the cell membrane (Figure 3d). The secretion of adenosine triphosphate (ATP) and high mobility group box 1 (HMGB‐1) protein, two well‐known DAMPs acting as “find me” signals,^[^ ^19^ ^]^ were also measured. The release of ATP showed a dose dependent manner in MNFs‐AA^−^ condition, reaching the highest ATP level at 80 µg mL^−1^ of MNFs, while AA^−^ media alone or MNFs‐Full were unable to stimulate ATP release (Figure 3e). MNFs‐AA^−^ also induced elevated secretion of HMGB‐1 particularly at concentration of 10 µg  mL^−1^ (Figure 3f). The reduced HMGB‐1 secretion at high concentrations is probably due to extensive cell death.^[^ ^20^ ^]^ The above results collectively indicate that the in situ immune response observed in Figure 2d is attributed to MNFs induced ICD of starved cancer cells, which should occur during the early stage post implantation with nutrient deficiency, rather than during cell culture in full media before implantation.

**Figure 4:**
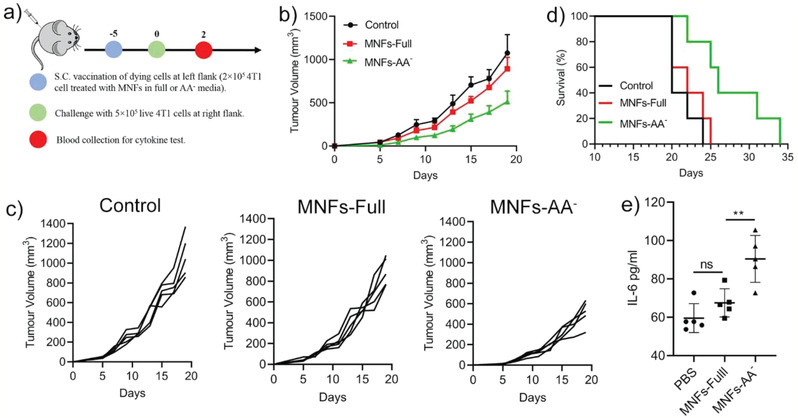
MNFs treated cancer cells in AA^−^ media function as cancer vaccines. a) Schematic illustration on the workflow. b,c) The overall and individual mouse tumor growth profile at the right flank (challenged with live 4T1 cells). d) The survival rate of mice vaccinated with MNFs treated cells in Full or AA^−^ media. e) IL‐6 level in serum on day 2. Data represent mean ± S.D. (*n* = 5). Unpaired *t*‐test was used to determine statistical differences. (*p < 0.05, **p < 0.01, ***p < 0.001).

To further validate the ICD‐inducing property of MNFs under nutrient deprived condition, a prophylactic vaccination model was performed. The 4T1 cells were treated in MNFs‐Full or MNFs‐AA^−^ condition at nanoparticles concentration of 80 µg mL^−1^ for 24 h, which led to a cell viability of ≤20% (Figure 2b). These dead/dying cells were subcutaneously inoculated at the left flank of BALB/c mice, and followed by live 4T1 tumor cells challenging 5 days later at the right flank (**Figure** **4**a). Whereas the vaccination with MNFs‐Full treated cells showed little prevention efficacy relative to control group (received PBS on day 5), the vaccination with MNFs‐AA^−^ treated cancer cells considerably delayed tumor grow profile at the right flank (Figure 4b,c), which was in agreement with the in vitro data on the capability of MNFs‐AA^−^ to induce ICD. The tumor prevention efficacy of MNF‐AA^−^ cells also resulted in an improved survival rate (Figure 4d). Moreover, the serum level of IL‐6, an important cytokine for T cell survival and proliferation, was significantly increased for the mice vaccinated with MNFs‐AA^−^ treated cells (Figure 4e). Interestingly, on day 18, 100% mice was found with a tumor at the left flank upon injection with MNFs‐Full treated cells that were used for vaccination, whereas MNFs‐AA^−^ treated cells only formed a tumor on one of five mice at the left flank (Figure S5, Supporting Information). All these results collectively indicate the generation of antitumor immunity by MNF‐AA^−^ treated cancer cells.

### 2.4 ICD Induction Mechanism

**Figure 5:**
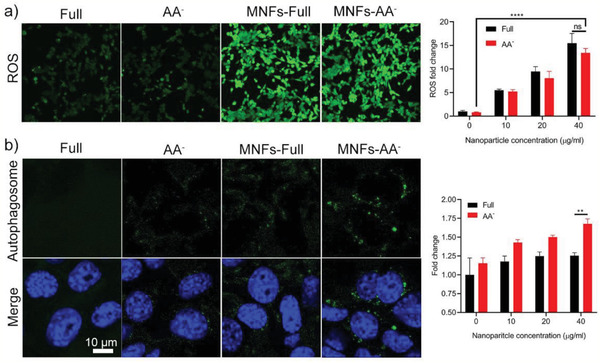
MNFs treatment in AA^−^ media concurrently induced elevated intracellular oxidative stress and autophagy. a) CLSM image on the intracellular oxidative stress as indicated by the fluorescent intensity of DCF‐DA and the corresponding quantitative results. b) CLSM image on the formation of autophagosome and the corresponding quantitative results. Data represent mean ± S.D. (*n* = 3). Unpaired *t*‐test was used to determine statistical differences (*p < 0.05, **p < 0.01, ***p < 0.001, ns: not significant).

Next, we sought to understand the underlying mechanism of the MNFs mediated ICD induction of cancer cells under nutrient‐deprived condition. It is well known that the intracellular oxidative stress is a major signaling pathway in eliciting ICD, as reported in a wide range therapeutic strategies, including chemotherapy, radiotherapy, and photodynamic therapy.^[^ ^3^, ^21^ ^]^ Therefore, the intracellular reactive oxygen species (ROS) induced by MNFs was investigated. As shown in CLSM image (**Figure** **5**a), compared to cells treated by Full or AA^−^ media, MNFs‐Full and MNFs‐AA^−^ treatment induced significantly enhanced 2′,7′‐dichlorofluorescin diacetate (DCF‐DA) fluorescent signal in a dose dependent manner, suggesting a greatly elevated intracellular ROS level. Quantitative data suggested that the MNFs‐Full and MNFs‐AA^−^ treatments respectively induced an ≈15.5‐ and ≈13.4‐fold increase in ROS level at nanoparticle concentration of 40 µg mL^−1^ compared to untreated cells, which can be attributed to the simultaneous GSH (an important intracellular antioxidant) depletion (Figure S7, Supporting Information) and Mn^2+^/Mn^4+^ mediated Fenton‐like reaction.^[^ ^22^ ^]^ However, it is noted that cells treated by MNFs‐AA^−^ produced comparable levels of ROS compared to those treated by MNFs‐Full, which is inconsistent with the MNFs‐AA^−^ induced enhancement of DAMP exposure as observed in Figure 3, implying the involvement of an undiscovered biological event in the promotion of ICD.

Autophagy is generally believed as a self‐defense mechanism to promote cell survival under extreme conditions.^[^ ^23^ ^]^ Although it has been reported that autophagy alone is insufficient to trigger ICD,^[^ ^24^ ^]^ the complicated interplay between autophagy and ICD has not been clearly understood so far. Recent studies have suggested that there might be an intimate correlation because i) the vesicles formed during autophagy (autophagosomes) are a source of tumor associated antigens; ii) autophagy can upregulate ATP release; iii) autophagy is a critical regulator of HMGB1 release during starvation.^[^ ^25^ ^]^ We deduced that the MNFs in nutrient deficiency might elicit the autophagy machinery that in turn contributes to induction of ICD. To test this hypothesis, an autophagy detection probe was employed, which can specifically accumulate in and stain autophagosome to evaluate the autophagic activity of cancer cells. As shown in Figure 5b, the formation of autophagosome surrounding the cell nuclei was visualized in the MNFs‐AA^−^ treated cells. Quantitative results reveal a dose‐dependent enhancement in the fluorescence emission intensity of the probe upon MNFs‐AA^−^ treated, reaching ≈1.7‐fold enhancement at the highest concentration compared to untreated cells in full media, indicative of elevated autophagic activity. In contrast, MNFs‐Full treatment was ineffective in promoting autophagy, despite their strong capability in inducing intracellular oxidative stress. Moreover, as shown in Figure S6, Supporting Information, the addition of chloroquine, a widely used autophagy inhibitor that blocks the binding of autophagosomes to lysosomes by inhibiting lysosomal acidification, greatly compromised the ICD as evidenced by the reduced secretion of HMGB‐1 (Figure S6a, Supporting Information). Moreover, 4T1 cells co‐treated with MNFs‐AA^−^ and chloroquine showed significantly reduced vaccine‐like activity in preventing the tumor growth (Figure S6b,c, Supporting Information). These results collectively suggest that the autophagic response is an important mechanism to the MNFs‐mediated ICD.

Based on these aforementioned results, it was speculated that the activation of ICD by MNF‐AA^−^ could be associated with the concurrently induced oxidative stress and autophagy, while solely promoting ROS (in the case of MNF‐Full) could only induce ICD to a very limited level (Figure 3). However, the biological mechanism of how the autophagic response was activated by MNF‐AA^−^ is still unclear. One possible explanation is related to the highly potent GSH depletion activity of MNFs particularly under the AA^−^ condition (Figure S7, Supporting Information) wherein the shortage of amino acid supply would reduce the intracellular GSH synthesis.^[^ ^26^ ^]^ GSH molecules have been reported to counteract autophagy by rendering intracellular protein‐thiols pool “resistant” to ROS‐mediated oxidation.^[^ ^27^ ^]^ Therefore, it is likely that MNFs with both GSH‐depleting and Fenton‐like reaction enabled ROS‐producing activities resulted in the markedly activated autophagy under starvation, although more systematic investigations and unambiguous evidences are required. It is worth mentioning that the autophagy study also offered a reasonable explanation to the cell viability data in Figure 2b. The pro‐survival role of autophagy effectively protected the cells from excessive oxidative damage,^[^ ^23^ ^]^ resulting in reduced cytotoxicity of MNFs in AA^−^ media than that in full media at high nanoparticle concentrations.

### 2.5 MNFs Orchestrated Cancer Starvation–ImmunoTherapy

**Figure 6:**
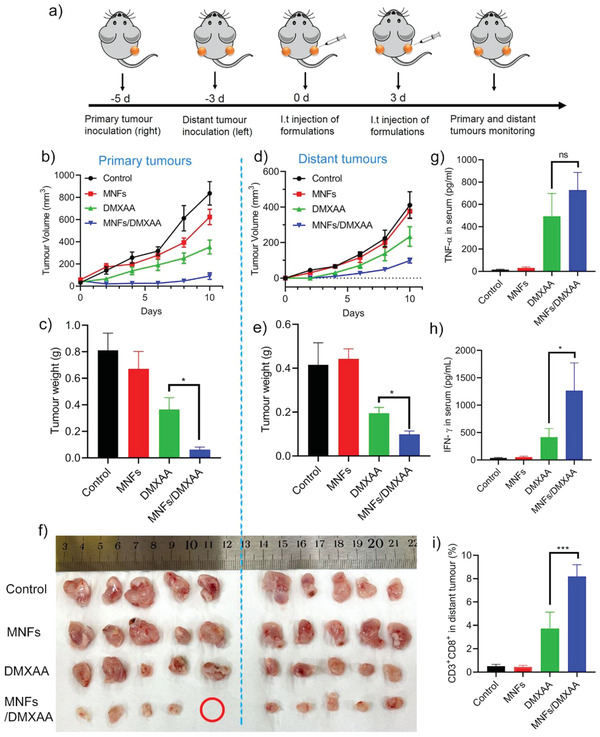
The in situ vaccination with MNFs/DMXAA provoked potent systemic antitumor immunity. a) Illustration of the workflow. b–f) Tumor‐growth curves, tumor weight, and optical images of tumors of mice treated with various formulations in a bilateral tumor model. The red circle represents the tumor that was completely eradicated by the treatment. g,h) The TNF‐*α* and INF‐*γ* levels in the serum measured on day 5. i) The infiltration of CD8+T cells (gated as CD3^+^ CD8^+^) in the distant tumor tissue. Data represent mean ± S.D. (*n* = 5). Unpaired *t*‐test was used to determine statistical differences (*p < 0.05, **p < 0.01, ***p < 0.001, ns: not significant).

To demonstrate the clinical significance of the findings on the unique nutrient‐responsive immunomodulatory activity of MNFs, it was hypothesized that those nanoparticles would be able induce antitumor immunity via a synergism with VDAs, a drug that specifically targets endothelial cells and damages the tumor vasculature system (blood vessels) to cut off nutrient supply to achieve tumor regression.^[^ ^9^ ^]^ DMXAA was chosen because it is not only a potent U.S. Food and Drug Administration approved VDA with high selectivity to tumor endothelial cells,^[^ ^28^ ^]^ but also an immune‐adjuvant that targets the stimulator of interferon genes (STING) pathway.^[^ ^29^ ^]^ These two pharmaceutical activities of DMXAA were expected to realize an ideal synergistic effect with MNFs: the vascular disrupting activity creates a nutrient deficient tumor microenvironment to activate the MNFs mediated ICD and antitumor immunity, which can be further improved by the immuno‐adjuvant activity. To test our hypothesis, a bilateral breast tumor model was established by subcutaneously implanting 4T1 cells into both the right and left flanks of BALB/c mice (**Figure** **6**a). The right tumors were designated as the primary tumors, which was subjected to treatments, while the left ones were designated as distant tumors that did not receive any treatments. When the primary tumors reached 30 to 50 mm^3^, various formulations were intratumorally injected. The MNFs/DMXAA nanoformulation for cancer starvation‐immunotherapy was prepared by simply mixing MNFs and DMXAA without pre‐encapsulation.

The combined MNFs/DMXAA treatment showed a greatly improved therapeutic efficacy in suppressing the primary tumor growth in comparison to DMXAA or MNFs alone (Figure 6b,c). Notably, the primary tumor on one of the five mice were completely eradicated (Figure 6f). Moreover, the MNFs/DMXAA treatment on primary tumors also resulted in a remarkable abscopal effect on inhibiting the growth of distant tumors located at the contralateral side (Figure 6d,e). However, DMXAA treatment on the primary tumors only moderately suppressed the progression of distant tumors due to a certain level of immune response resulted from its intrinsic immuno‐stimulating activity, while the growth of distant tumors in MNFs group was not significantly affected. The trend of CD8^+^ T cell infiltration in distant tumors agreed well with the tumor growth profile (Figure 6i). The MNFs/DMXAA group showed a substantial amount of CD8^+^ T cell recruited within the distant tumors. The DMXAA treatment only resulted in a moderate CD8^+^ T cell infiltration efficiency, and this infiltration levels within distant tumors in MNFs and control groups were negligible. Furthermore, the level of TNF‐*α* and IFN‐*γ*, two important cytokines in cell mediated immunity, in mice sera was measured by enzyme‐linked immunosorbent assay (ELISA). It was revealed that the in situ vaccination with MNFs/DMXAA induced the highest level of TNF‐*α* and IFN‐*γ* secretion in serum among all the groups (Figure 6g), indicative of a robust antitumor immunity (Figure 6g,h). Importantly, the combined MNFs/DMXAA therapy induced dramatic changes in the tumor microenvironment, including enhanced secretion of pro‐inflammatory cytokines (Figure S8a–c, Supporting Information), increased infiltration of white blood cells (CD45^+^), CD4^+^ T Cells (CD4^+^), CD8^+^ T Cells (CD8^+^) and dendritic cells (CD11c^+^) (Figure S8d, Supporting Information), activation of cytotoxic T lymphocyte (CD8^+^ IFN‐*γ* ^+^) (Figure S8e, Supporting Information), maturation of dendritic cells (CD11c^+^CD86^+^) (Figure S8f, Supporting Information), and enhanced CRT expression and HMGB‐1 secretion. These results strongly suggest the synergistic effect of MNFs and DMXAA in provoking strong ICD and systemic antitumor immunity to suppress both primary and distant tumors.

To gain further evidence on the antitumor mechanism, immunohistology analysis of both primary and distant tumors from different groups were performed. As revealed by immunohistochemical staining with CD31 antibody (Figure S9a, Supporting Information), the primary tumors in the control and MNFs group were highly vascularized. In contrast, the tumors resected from DMXAA or MNFs/DMXAA treated mice were only sparsely vascularized and the sizes of blood vessels were reduced, suggesting a potent vascular disrupting activity of DMXAA, which led to cancer starvation and thus extensive necrosis of cancer cells as revealed by H&E staining (Figure S9b, Supporting Information). Moreover, the primary tumors from MNFs/DMXAA group revealed a substantial amount of CD8 T cells infiltration, indicative of strong antitumor immunity (Figure S9c, Supporting Information). Notably, MNFs/DMXAA also resulted in significantly enhanced infiltration of CD8 T cells in the distant tumors compared to all other groups (Figure S10a, Supporting Information), consistent with the flow cytometry data (Figure 6i), which led to extensive cell apoptosis as revealed by the terminal deoxynucleotidyl transferase dUTP nick end labeling (TUNEL) staining (Figure S10b, Supporting Information). In addition, MNFs/DMXAA treatment did not obviously affect the body weight of mice (Figure S11, Supporting Information), and no acute damage in major organs was observed as shown by H&E staining (Figure S12, Supporting Information), indicative of good biocompatibility of this new therapeutic strategy.

## 3 Conclusion

In summary, we have fabricated PEGylated MNFs and demonstrated their unprecedented immunomodulatory activity in augmenting ICD in response to nutrient deficiency. This finding represents a new property of MnO_2_ nanoparticles that can promote immuno‐surveillance and systemic immune response to eradicate both local and distant tumors. MNFs have the potential to be used as a unique ICD inducer compared to reported ones from two aspects. First, distinct from conventional ICD inducers that are small molecules (such as doxorubicin and oxaliplatin), MNFs are in a particulate form, which allow them to encapsulate and deliver additional therapeutics, either small molecules or biomacromolecules, to fulfill diverse sophisticated biomedical designs. Second, conventional molecular ICD inducers generally possess low targeting activity, thus comparably affect various types of cells. In contrast, the ICD‐inducing capability of MNFs is selective to starved cells, which offers the advantage of minimizing the side effects to normal tissues, if nutrient deficiency is selectively induced in tumors through, for instance, VDAs. The potent antitumor activity of the combination of MNFs and DMXAA further suggests a novel nanotherapeutic paradigm, that is, nanoparticle orchestrated cancer starvation‐immunotherapy. Future works should focus on optimizing the structural properties of MnO_2_ nanoparticles, such as surface textures (rough or smooth), which have been reported as an important parameter in dictating their immunomodulatory activity.^[^ ^30^ ^]^ Furthermore, the combination of immune checkpoint blockades, such as PD‐1/PD‐L1 antibody with the MNFs/DMXAA formulation is worth of investigation in the future, which is expected to further improve the antitumor performance. It is envisioned that the fundamental finding on the immunomodulatory property of MnO_2_ nanoparticles would inspire vast elegant designs of sophisticated nanomedicines, and the intriguing therapeutic paradigm would have the potential to be developed as an in situ vaccination in neo‐adjuvant settings for clinic use.

## Conflict of Interest

The authors declare no conflict of interest.

## Supporting information

Supporting Information
