# Supplementary material for: MnO2 Nanoflowers Induce Immunogenic Cell Death under Nutrient Deprivation: Enabling an Orchestrated Cancer Starvation‐Immunotherapy
Source: Adv Sci (Weinh). 2020 Dec 31;8(4):2002667. doi: 10.1002/advs.202002667 (PMC7887587; doi:10.1002/advs.202002667)
Supplement: Supplementary file 1 — Supporting Information [file ADVS-8-2002667-s001.pdf]

© 2020 Wiley-VCH GmbH

ADVANCED SCIENCE
Open Access

Supporting Information

for *Adv. Sci.*, DOI: 10.1002/advs.202002667

# $MnO_2$ Nanoflowers Induce Immunogenic Cell Death under Nutrient Deprivation: Enabling an Orchestrated Cancer Starvation-Immunotherapy

*Yannan Yang, Zhengying Gu, Jie Tang, Min Zhang, Yang Yang, Hao Song, and Chengzhong Yu**

# Supporting Information

**$MnO_2$ Nanoflowers Induce Immunogenic Cell Death under Nutrient Deprivation: Enabling an Orchestrated Cancer Starvation-Immunotherapy**

*Yannan Yang, Zhengying Gu, Jie Tang, Min Zhang, Yang Yang, Hao Song, Chengzhong Yu**

**Synthesis and PEGylation of $MnO_2$ nanoflowers (MNFs).** MNFs were synthesized via a facile one pot strategy. Briefly, 5 mL of $KMnO_4$ stock solution (2 mg/mL) were added into a plastic container containing 5 mL of MES buffer (100 mM, pH 6.0) and 2 mL of water at room temperature (~ 25 °C). The resultant homogeneous solution was sonicated for 30 min until dark-brown precipitates were formed. MNFs were collected by centrifugation (5000 × g) and washed three times with deionized water.

For PEGylation, 24 mg of PEG-$NH_2$ (MW 5k) was dissolved in a solution containing 5.7 mL ethanol and 300 μL $H_2O$ to make a stock solution with PEG concentration of 4 mg/mL. 1 mg of MNFs were dispersed in 3 mL of ethanol and followed by addition of 1 mL PEG stock solution. The resultant MNFs dispersion was stirred at room temperature for 48 h to allow sufficient PEGylation.

**Cell culture.** 4T1 cells (ATCC) were cultured in RPMI1640 (Gibco) supplemented with 10% fetal bovine serum (FBS, Sigma) and 1% penicillin/streptomycin (Gibco). Heat-inactivated FBS was prepared by heating at 56 ± 2°C water bath for 30 mins and filtered at 4°C to remove precipitates. Amino-acid-free medium was prepared by centrifuging heat-inactivated FBS in centrifugal filters (MWCO 10K, Amicon Ultra-4, Merck) and addition to base media without amino acids (mybiosource).

**Reagents.** 2-(N-morpholino)ethanesulfonic acid (MES), Potassium permanganate (KMnO4) and 2’ ,7’-Dichlorofuorescein diacetate (DCFH-DA) were purchased from Sigma Aldrich. Autophagy detection Kit were purchased from Enzo Life Sciences. GSH detection Kit was

purchased from Cell Signalling Technology. Alexa Fluor® 488 conjugated anti-Calreticulin (CRT) antibody (ab196158), Alexa Fluor® 647 conjugated anti-Hsp70 antibody (ab204691) and Alexa Fluor® 488 conjugated anti-Hsp90 antibody (ab223647) were purchased from Abcam. IFN-γ, TNF-α and IL-6 ELISA kit were purchased from Abcam. Alexa Fluor® 488 conjugated Anti-CD3, APC conjugated anti-CD8 antibodies were purchased from Biolegend. DMXAA (5,6-Dimethylxanthenone-4-acetic Acid) was purchased from Sigma-Aldrich. All chemicals were used as received from the suppliers without further purification. Ultrapure water (18.2MΩ; Millpore Co., USA) was used throughout the experiment.

**In vitro cytotoxicity assay.** MTT assay was used to determine the cytotoxicity of MNFs in Full or $AA^-$ culture media. Briefly, 4T1 cells were seeded into 96-well plates (100 μL) in Full or AA- media, and were cultured at 37 °C with 5% $CO_2$. For 24 h test, the cell density were $8\times10^3$/well in full media and $1.6\times10^4$/well for $AA^-$ media. For 6 h test, the cell density were $2\times10^4$/well for both media. After 24 h incubation, different concentrations of MNFs were added to the wells. The cells were subsequently incubated for 6 or 24 h. Then, 10 μL of MTT solution (5 mg $mL^{-1}$) was added to each well and the plate was placed in the incubator for additional 4 h. The media was removed and DMSO (100 μL) was added into each well. Absorbance values were determined with Bio-Rad model-680 microplate reader at 570 nm.

**Determination of ROS generation in vitro.** For ROS detection, $2\times10^5$ 4T1 cells were seeded in six-well plates in full media. After 24 h, the original media was removed, and cells were incubated with MNFs (40 μg $mL^{-1}$) in full or $AA^-$ media for 6 h. The treated cells were washed with PBS twice and incubated with 10 μM of DCFH-DA (diluted from 10 mM DCFH-DA stock solution in Dimethyl sulfoxide) in serum free RPMI1640 for 30 min. The unloaded probe was removed by washing once with PBS, and fresh PBS was added into each well. The fluorescence intensity of cells was imaged by confocal microscopy. The ROS intensity was quantified by microplate reader. The protocol was similar to the imaging

protocol as described above, with the only difference that $1\times10^4$ 4T1 cells was seeded in 96-well black plate with transparent bottom.

**Autophagy detection.** 4T1 cancer cells were seeded in black 96-well plates at a density of 8,000 cells/well in full media. After 24 h, the original medium was removed and various concentration of MNFs in full or $AA^-$ media were added and incubated for 6 h. The autophagy detection reagent (Enzy Life Sciences) and Hoechst 33342 was added according to the manufacture's protocol and the fluorescent intensity was measured using the microplate reader. The autophagy detection reagent fluorescent intensity was normalized using Hoechst 33342 fluorescent intensity. For confocal imaging of autophagosome, 4T1 cancer cells were seeded 6-well plates at a density of $2\times10^5$ cells/well in full media. After 24 h, the original medium was removed and 40 µg/mL of MNFs in full or $AA^-$ media were added and incubated for 6 h. The Autophagy detection reagent and Hoechst 33342 was added according to the manufacture's protocol. The cells were then fixed with 4% paraformaldehyde for 30 min at 4 °C, washed and imaged using confocal microscopy.

**Cell uptake:** To quantitatively compare cellular uptake of MNFs, $2\times10^5$ 4T1 cells per well were seeded in a 6-well plate and cultured in Full or $AA^-$ media for 24 h. Cells were then incubated with MNFs (40 µg/mL) in Full or $AA^-$ media for 6 or 24 h followed by washing with PBS and harvested with trypsin. After centrifugation, the cell pellets were washed twice with PBS before cell number counting. The cells were then lysed by cell lysis buffer (Cell Signaling Technology) and the supernatants (containing cell components) were removed by centrifugation at 13,000 rpm for 10 minutes, followed by two washes with PBS. Aqueous HCl solution (1 M) was then added to allow dissolution of the nanoparticles with ultrasound sonication. The Mn concentrations in the final solutions were measured by inductively coupled plasma optical emission spectrometer (ICP-OES) with a Vista-PRO instrument (Varian Inc, Australia). All experiments were performed in triplicate.

**Intracellular GSH measurement.** 4T1 cancer cells were seeded in black 96-well plates at a density of 8,000 cells per well in full media. After 24 h, the original medium was removed and various concentrations of MNFs in full or $AA^-$ media were added and incubated for 6 h. The cellular GSH probe (Cell Signaling Technology) was then added according to the manufacture's protocol and the fluorescent intensity was measured using the microplate reader.

**In vitro ecto-CRT, ecto-Hsp70 and ecto-Hsp90 detection.** Ecto-CRT exposure induced by MNFs was evaluated by flow cytometry and confocal laser scanning microscopy (CLSM). For flow cytometry analysis, 4T1 cells were seeded in 6-well plate at density of $1\times10^5$/well and incubated for 24 h in full media. Subsequently, the original culture media was remove and the cells were treated with full media, $AA^-$ media, or MNFs (80 μg $mL^{-1}$) in full media or $AA^-$ media for 24 h. Then the cells were washed, harvested with trypsin, and incubated with Alexa Fluor 488-CRT antibody (1/500 dilution) for 50 min at 4 °C. The cells are incubated in 500 μL PBS containing 10 μg/mL propidium iodide before assessment in a flow cytometer. For CLSM analysis, cells were incubated with Alexa Fluor 488-CRT antibody for 50 min at 4 °C, fixed with Paraformaldehyde (4%), stained with DAPI, and observed under CLSM. For ecto-Hsp70 and ecto-Hsp90 detection, the protocol was similar to ecto-CRT detection, except that Alexa Fluor 488-Hsp90 (1/1000 dilution) and Alexa Fluor 647-Hsp70 (1/1000 dilution) were used for staining.

**ATP and HMGB-1 release.** For ATP release test, cells were seeded into 12-well plates at the density of $1\times10^5$ cells/per well in full media and incubated for 24 h. Then the original culture media was removed and the cells were treated with MNFs dispersed in full or $AA^-$ media at various concentrations for 24 h. Full and $AA^-$ culture media were used as control. Cell supernatant was collected and tested in ATP Luminescence Assay Kit (A22066, ThermoFisher) following the manufacture's protocol. The luminescence was measured by a microplate reader (Synergy Mx, BioTeK). For HMGB-1 release test, cell supernatant was

collected after 24 h incubation and tested in an HMGB-1 ELISA kit (Novus Biologicals) according to the manufacturer's instructions. To investigate the role of autophagy, chloroquine (50 μM) and MNFs (10 μg/ml) were used to treat cells in Full or $AA^-$ media. The following procedures were the same as described above.

**Animals.** Female BALB/c (6~8 weeks of age) mice were purchased Biological Resource Facility, The University of Queensland. All experiments were approved by The University of Queensland, and were carried out in accordance with the institutional guidelines for animal experimentation.

***In vivo* tumorigenicity of MNFs treated cancer cells.** 4T1 cells were treated with PBS or 10 μg/mL MNFs in full culture media for 6 h. Subsequently, $2\times10^6$ treated 4T1 cells were injected into the right flank of 6 week old female BALB/c mice (n=5) on day 0. On day 10, blood was collected and the serum was used for measuring TNF-α and IFN-γusing Elisa kit (Abcam). The size of tumours was measured by a digital caliper every other day, and the tumour volume was calculated using the formula $V = (\text{length})\times(\text{width})^2/2$.

***In vivo* vaccination with dying cells.** 4T1 cells were exposed to PBS, 80 μg/mL MNFs in full or $AA^-$ culture media for 24 h. The dying $2\times10^5$ 4T1 cells were injected into the left flank of 6 week old female BALB/c mice (n=5) on day 0. On day 5, the animals received subcutaneous injection of $5\times10^5$ live 4T1 cells in the contralateral (right) flank. Blood was collected on day 2 and the serum was used for measuring IL-6 using Elisa kit (Abcam). The size of tumours was measured by a digital caliper every other day, and the tumour volume was calculated using the formula $V = (\text{length})\times(\text{width})^2/2$. The survival of mice was recorded and the death was defined as the tumour size exceed 1000 $mm^3$ or severe ulceration was observed according to the institutional animal ethics.

For the *in vivo* vaccination with dying cells pre-treated with autophagy inhibitors, the procedure was the same as described above, except that 4T1 cells were exposed to chloroquine (50 μM) and MNFs in $AA^-$ media.

***In vivo* anti-tumour assessment in bilateral tumour model.** $2\times10^6$ 4T1 cells were suspended in PBS and subcutaneously injected in the right flank of BALB/c mice on day -5 (primary tumours) and $5\times10^5$ 4T1 cells were subcutaneously injected in the left flank on day -3 (distant tumours). On day 0, the tumours at right flank (primary tumours) were intratumourally injected with 30 µl of Saline, MNFs (7.5 mg $kg^{-1}$), DMXAA (7.5 mg $kg^{-1}$) or a mixture of MNFs (7.5 mg $kg^{-1}$) and DMXAA (7.5 mg $kg^{-1}$) on days 0 and day 2. Eye bleed was conducted on day 5 to test the levels of TNF-α and IFN-γ in the serum using ELISA. The mice were euthanized on day 10 and the distant tumours were collected to analyse the infiltration of T cells.

**Flow cytometry analysis.** Tumors were removed and minced with scissors prior to incubation with 1 mg/ml collagenase A and 0.2 mg/ml DNase I (Sigma-Aldrich) for 30 min at 37 °C. The digested tissues were gently grinded with rubbers on syringes and meshed though a 70 µM cell strainer to obtain single cells suspension. Infiltrating immune cells counts were normalized by tumour mass. The single-cell suspensions were washed with PBS and resuspended in cell staining buffer (BioLegend). Following cell counting and aliquoting in 1.5 ml vials, cells were stained with dye-conjugated antibodies including anti-CD3, anti-CD4, anti-CD8α, anti-CD45, anti-CD11c, anti-CD86 and anti-CRT for 20 min at 4 °C. For intracellular staining, cell surface antigen staining was firstly performed, and cells were fixed in 4% PFA and permeabilized in 0.1% Tween/PBS in the dark for 20 min at room temperature. Then these cells were resuspended in PBS containing 10% normal goat serum and 0.3M glycine to block non-specific protein binding, and stained with TNF-α and IFN-γ antibodies. The cell number of expression of cell surface markers was analysed using flow cytometer.

**Intratumoural cytokine analysis.** Tumours were homogenized in T-PER™ Tissue Protein Extraction Reagent (Thermal Scientific) supplemented with Halt Protease Inhibitor Cocktail

(Thermal Scientific). The samples were then centrifuged and supernatant were collected for ELISA test.

**Statistical analysis.** Data are presented as the mean ± standard deviation (s.d.) based on at least three independent experiments or specified in each experiment. The statistical analysis was performed using Graphpad Prism 8 software. Unpaired Student's two-sided t test analysis was used for comparing the difference between two groups. One-way analysis of variance (ANOVA) was used for comparing the difference among multiple groups. Asterisks indicate significant differences (*$P < 0.01$, **$P < 0.005$, ***$P < 0.001$).

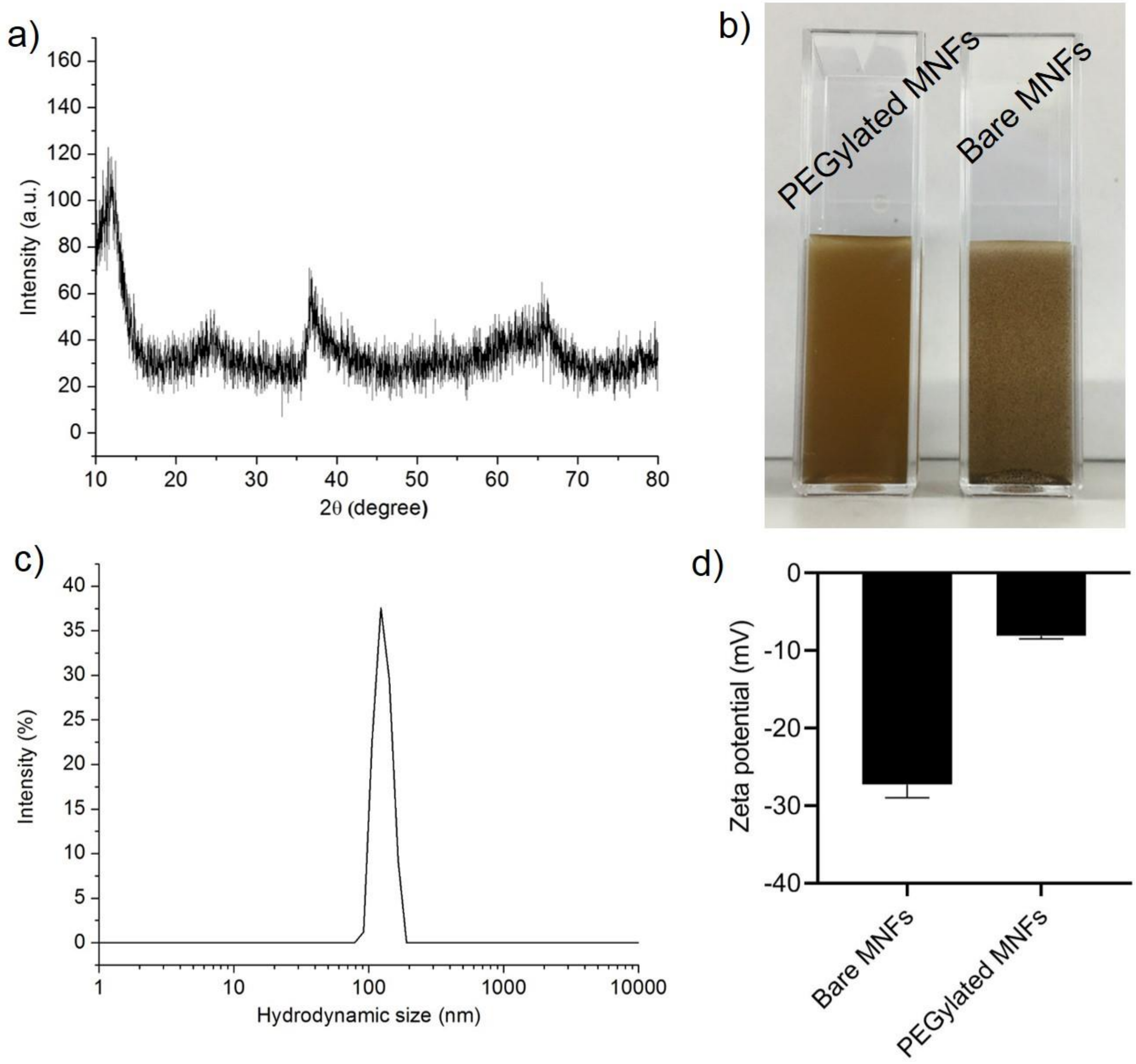


Figure S1. a) XRD pattern of as prepared MNFs. b) Optical image of bare MNFs and PEGylated MNFs suspension in PBS. c) DLS result of PEGylated MNFs in PBS. d) Zeta potential of MNFs before and after PEGylation in PBS.

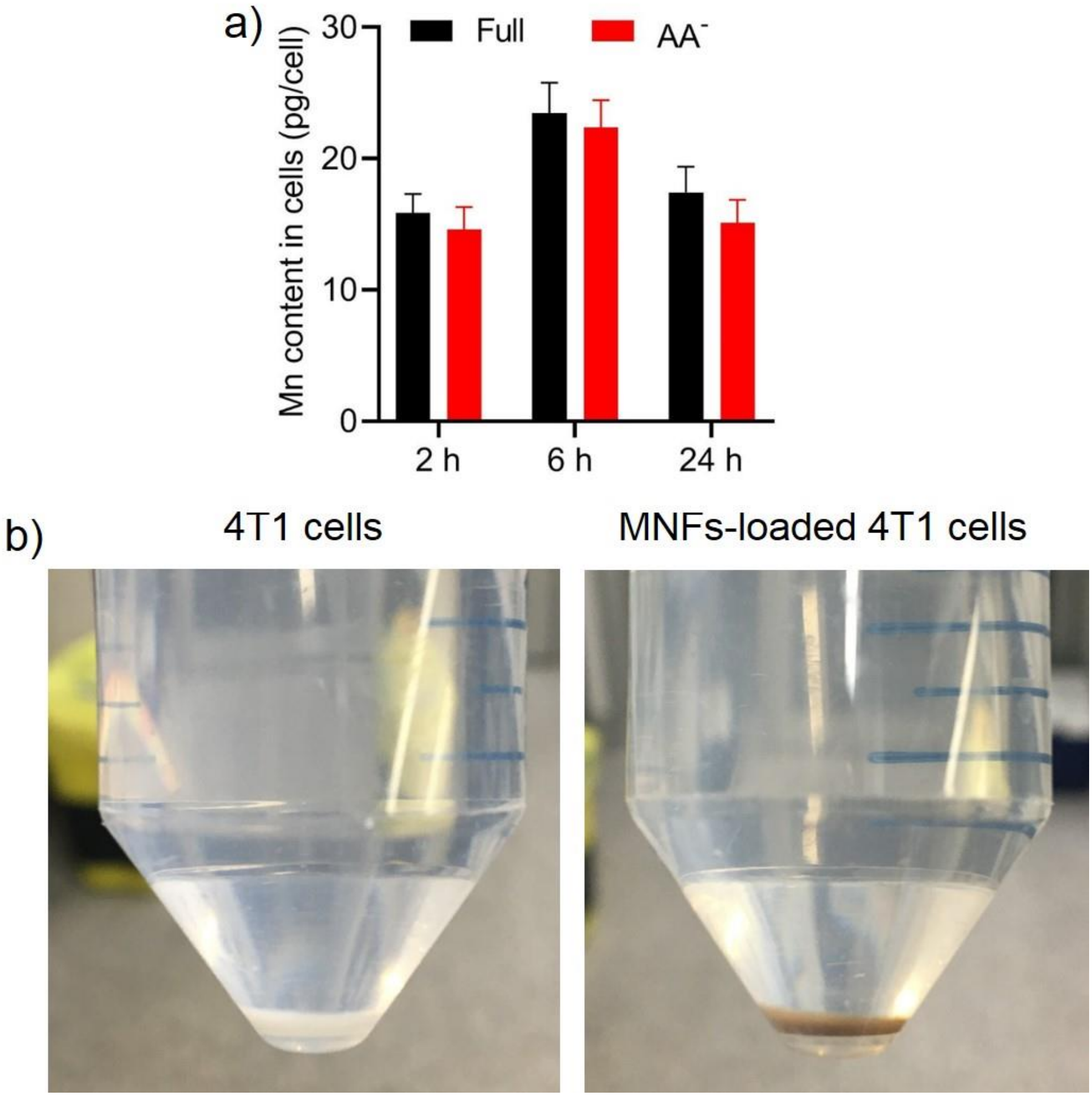


Figure S2. a) Time dependant cellular uptake of MNFs in 4T1 cells as indicated by intraocular Mn content measured by ICP-OES. b) Optical images of 4T1 cells and MNFs-loaded 4T1 cells. Data represent mean ± s.d. (n = 3).

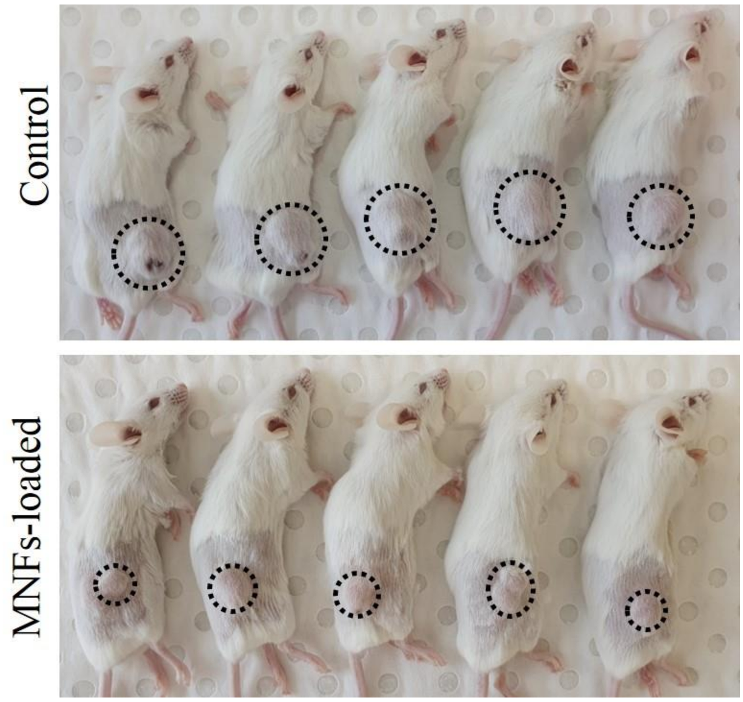


Figure S3. Optimal images of tumour formed in mice (day 18) inoculated with control 4T1 cells or MNFs-loaded 4T1 cells.

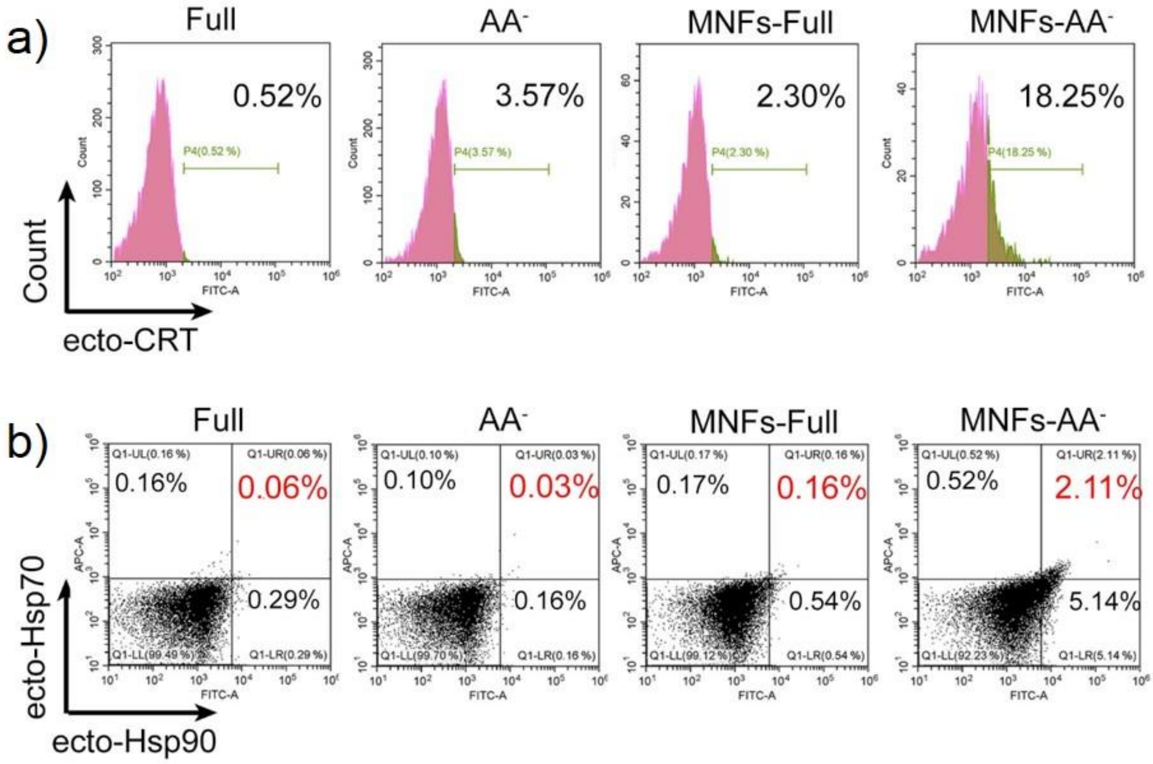


Figure S4. Flow cytometry data of surface exposure of a) ecto-CRT, b) ecto-Hsp70 and ecto-Hsp90 on 4T1 cells.

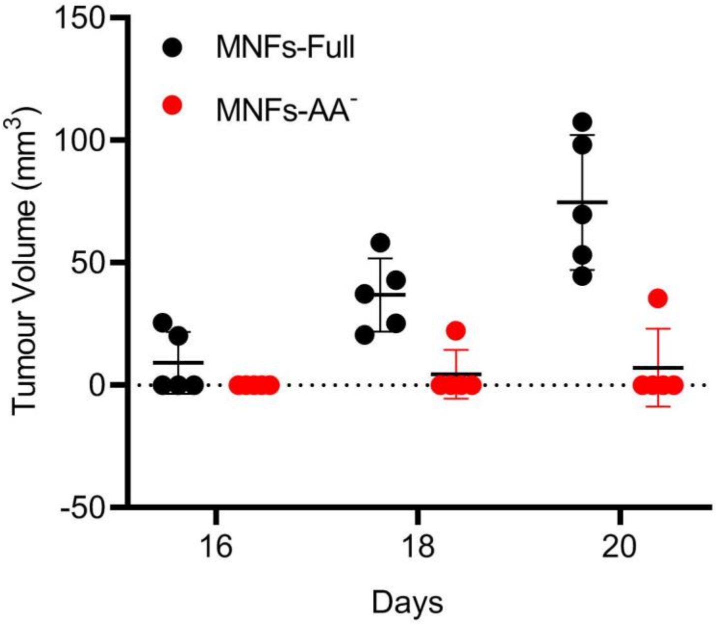


Figure S5. Tumorigenesis of MNFs-Full or MNFs-AA$^-$ treated cells at left flank. In the prophylactic vaccine model, MNFs-Full treated 4T1 cancer cells formed tumours at the left flank of all the mice in this group on day 18, while MNFs-AA$^-$ treated 4T1 cancer cells only formed a tumour on 1/5 of mice in this group. Data represent mean ± s.d (n = 5).

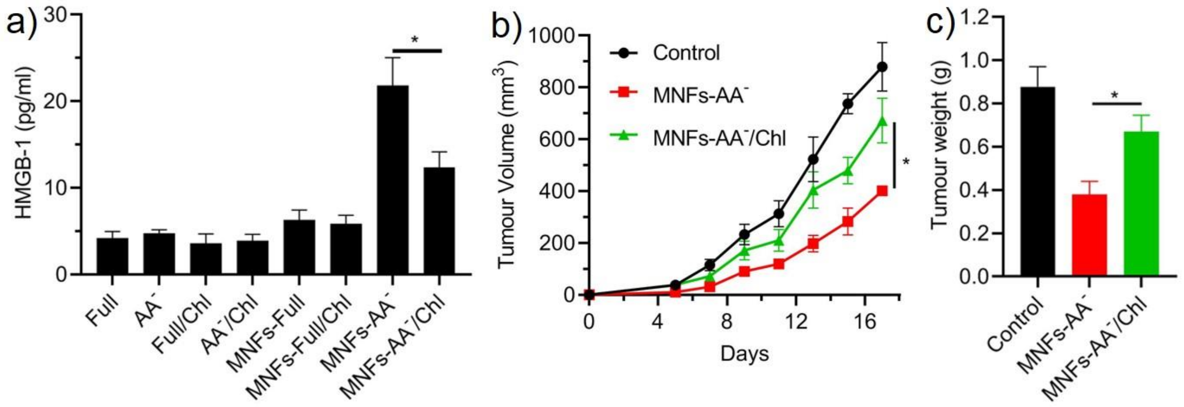


Figure S6. Autophagy inhibitor reduced the MNFs mediated ICD under nutrient deficiency. a) Secretion of HMGB-1 in the cell culture media. b, c) The overall tumour growth profile and tumour weight on day 17. 4T1 cells were treated with MNFs (80 µg/ml) or MNFs (80 µg/ml) + Chl (50 µM) in AA$^-$ media for 24 h, and subcutaneously injected into left flank of mice as vaccines on day -5. On day 0, mice were challenged with live cells and the tumor growth were monitored. For the control group, the mice did not receive any vaccination before challenging with live cells. Data represent mean ± s.d. (n = 4). One-way ANOVA (for b) and unpaired T test (for a, c) were used to determine statistical differences (*$p$ < 0.05, **$p$ < 0.01, ***$p$ < 0.001).

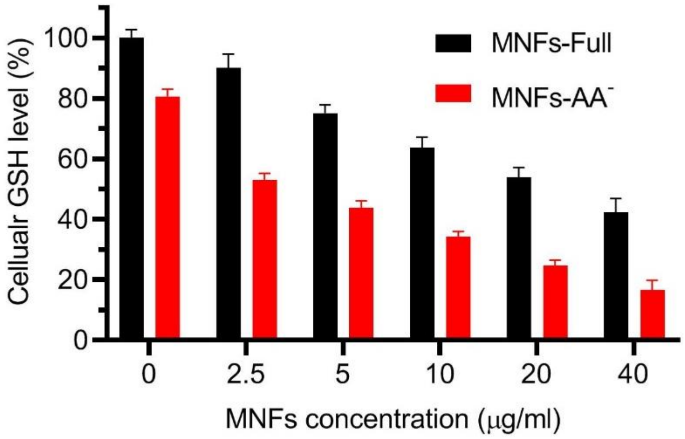


Figure S7. MNFs mediated intracellular GSH depletion after treatment of 4T1 in full or AA$^-$ culture media for 6 h. Data represent mean ± s.d. (n = 4).

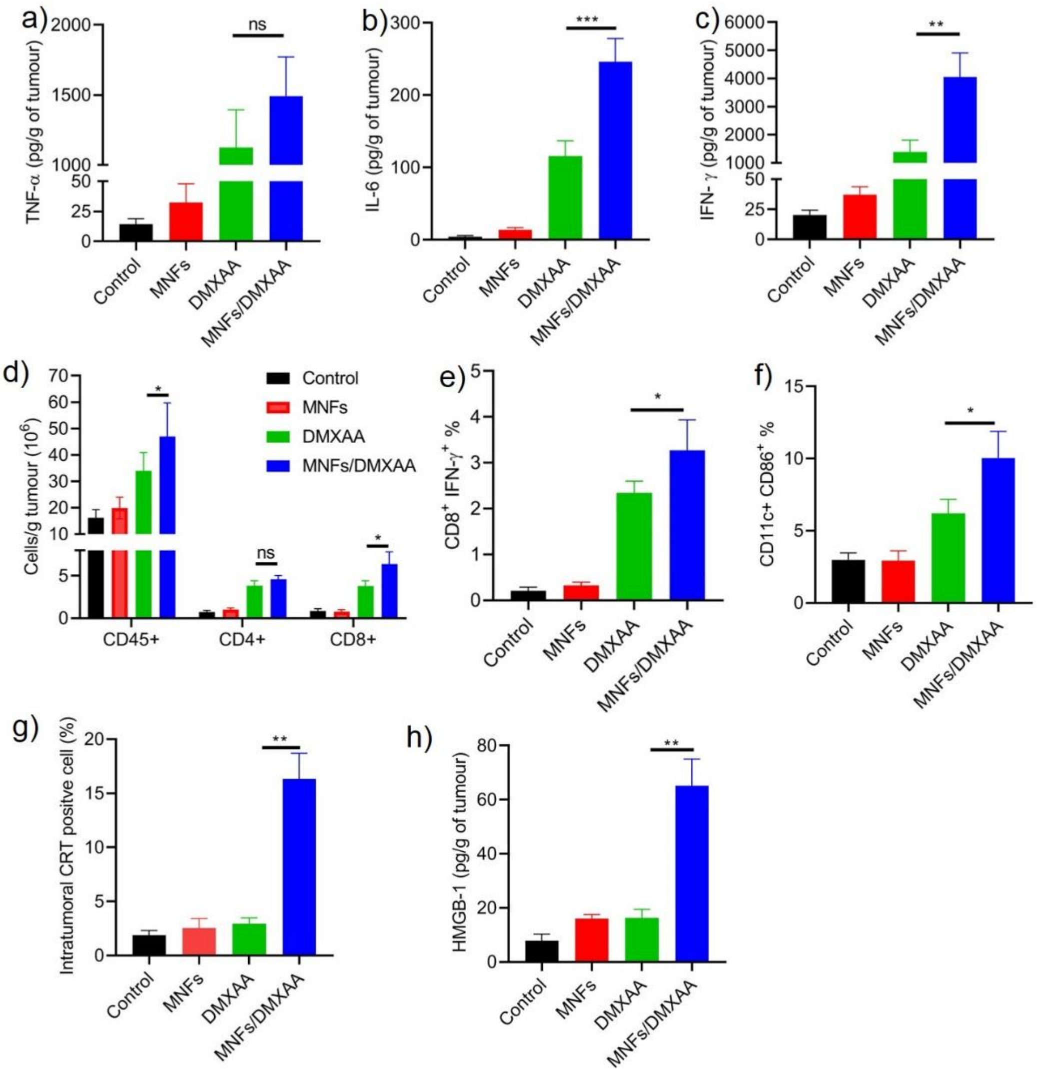

Figure S8. MNFs/DMXAA induced dramatic changes in the tumour microenvironment. a-c) Intratumoral cytokine level. d) Absolute numbers of immune cells. e) The infiltration of CTLs (defined as $CD8^{+}IFN\text{-}\gamma^{+}$) in tumours. f) Maturation of dendritic cells in tumours. g) CRT positive cells in tumours. h) Intratumoral HMGB-1 level. Tumours were inoculated on day -5, and received various formulations in day 0. Tumour were harvested on day 5 for analysis. Data represent mean ± s.d. (n = 3). Unpaired T test was used to determine statistical differences (*$p < 0.05$, **$p < 0.01$, ***$p < 0.001$, ns: not significant).

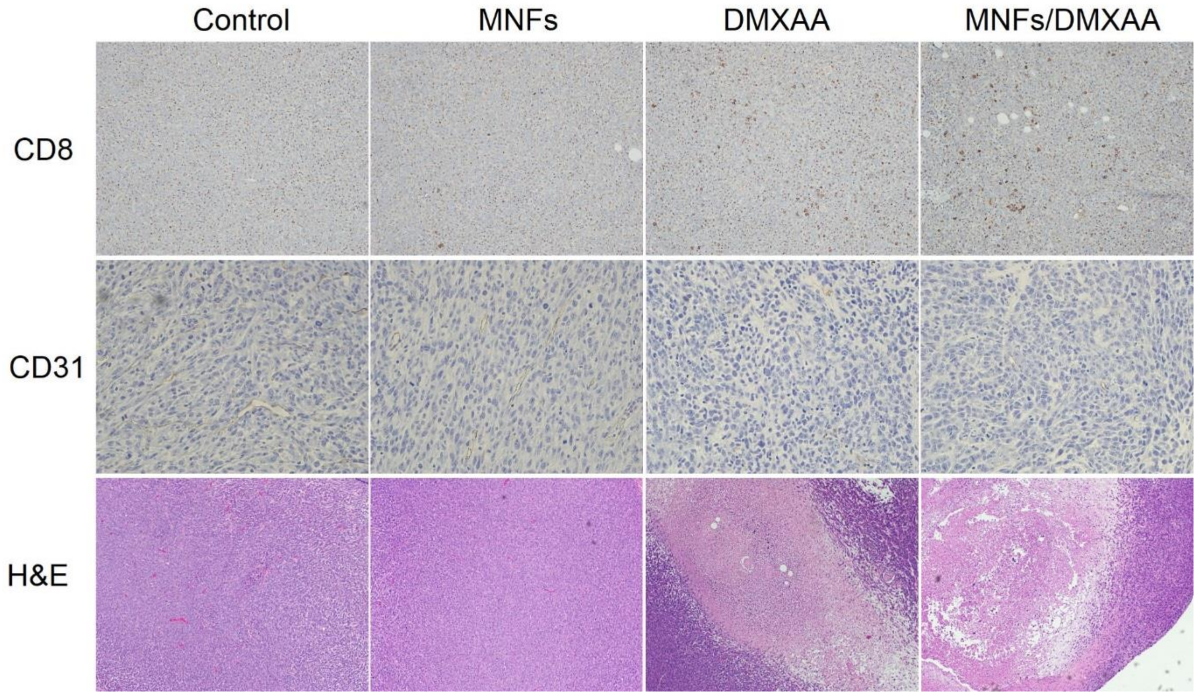


Figure S9. Immunohistochemical and H&E analysis of primary tumours in bilateral tumour model.

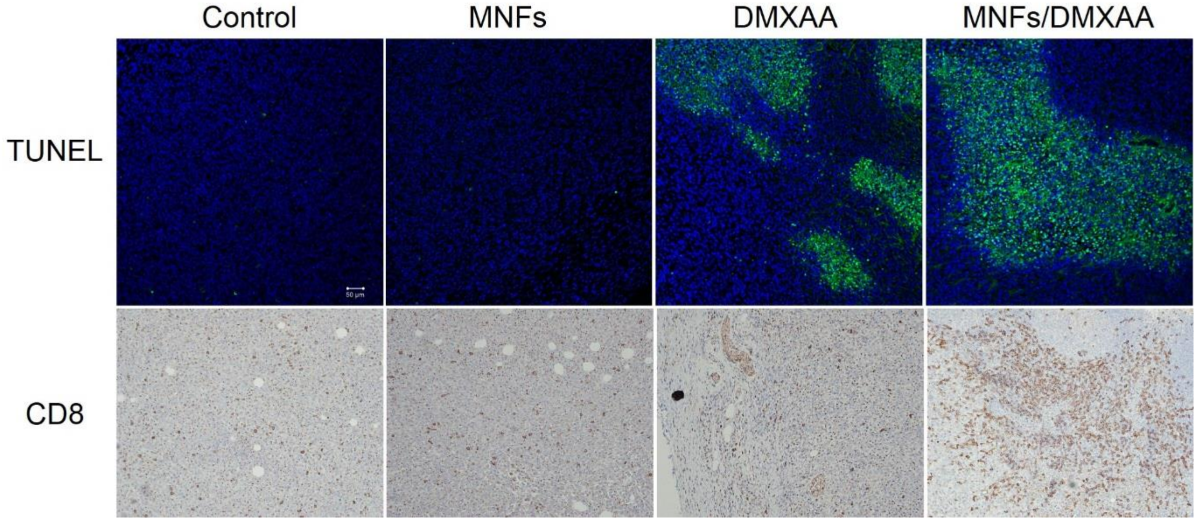


Figure S10. TUNEL and Immunohistochemical analysis of distant tumours in bilateral tumour model.

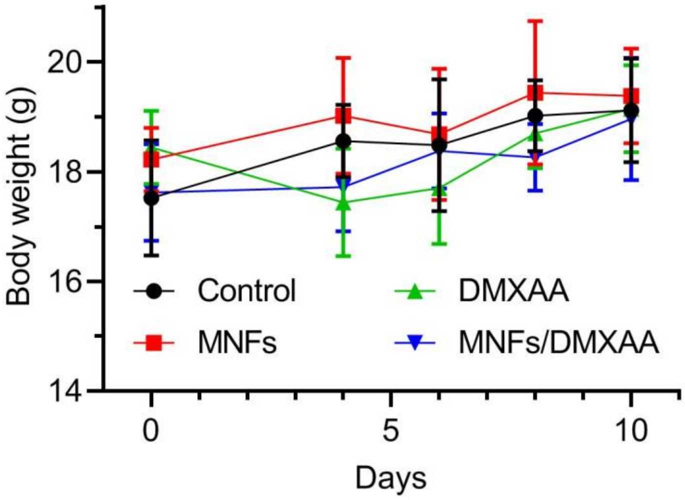


Figure S11. Body weight of BALB/c mice in bilateral tumour model. Data represent mean ± s.d. (n = 5).

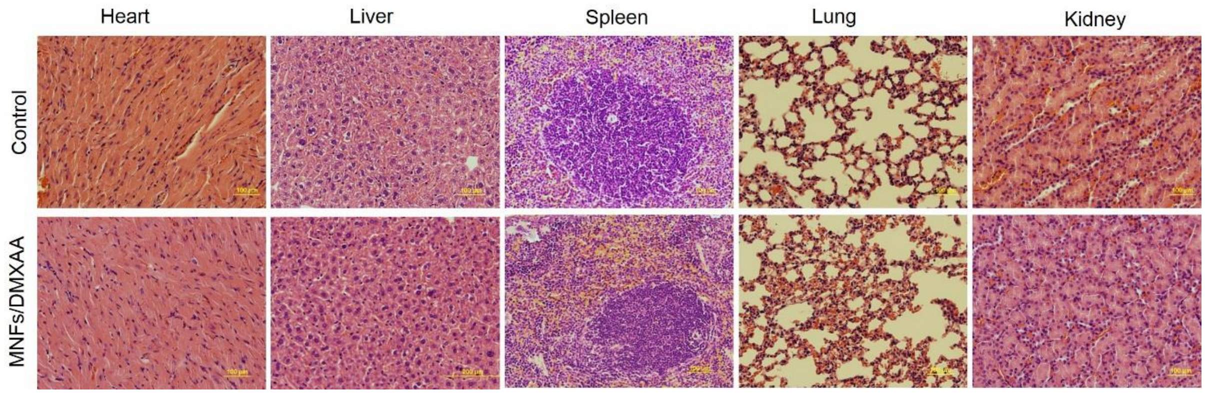

Figure S12. Representative H&E staining images of major organs of mice administrated with PBS (Control) or MNFs/DMXAA (MNFs: 7.5 mg $kg^{-1}$, DMXAA: 22.5 mg $kg^{-1}$).
